# A novel tool-use mode in animals: New Caledonian crows insert tools to transport objects

**DOI:** 10.1007/s10071-016-1016-z

**Published:** 2016-07-20

**Authors:** Ivo F. Jacobs, Auguste von Bayern, Mathias Osvath

**Affiliations:** 1 Department of Cognitive Science, Lund University, Helgonavägen 3, 22100 Lund, Sweden; 2 Department of Behavioural Ecology and Evolutionary Genetics, Max Planck Institute for Ornithology, Eberhard-Gwinner-Straße, 82319 Seewiesen, Germany; 3 Department of Zoology, University of Oxford, South Parks Road, OX1 3PS Oxford, UK

**Keywords:** Tool use, New Caledonian crow, Play, Object exploration, Tool transport

## Abstract

New Caledonian crows (*Corvus moneduloides*) rely heavily on a range of tools to extract prey. They manufacture novel tools, save tools for later use, and have morphological features that facilitate tool use. We report six observations, in two individuals, of a novel tool-use mode not previously reported in non-human animals. *Insert*-*and*-*transport* tool use involves inserting a stick into an object and then moving away, thereby transporting both object and tool. All transported objects were non-food objects. One subject used a stick to transport an object that was too large to be handled by beak, which suggests the tool facilitated object control. The function in the other cases is unclear but seems to be an expression of play or exploration. Further studies should investigate whether it is adaptive in the wild and to what extent crows can flexibly apply the behaviour in experimental settings when purposive transportation of objects is advantageous.

**Electronic supplementary material:**

The online version of this article (doi:10.1007/s10071-016-1016-z) contains supplementary material, which is available to authorized users.

## Introduction

New Caledonian crows (*Corvus moneduloides*) are habitual and proficient tool users. They use and manufacture tools in several modes in both natural settings and captivity (animal tool use is reviewed in Bentley-Condit and Smith 2010; McGrew 2013; Shumaker et al. 2011, with additional modes in New Caledonian crows described by Jelbert et al. 2014; Taylor et al. 2012; Troscianko et al. 2008; von Bayern et al. 2009). A large portion of their daily caloric intake may be obtained through tool use (Rutz et al. 2010). Their reliance on tools has possibly resulted in morphological adaptations: a wide binocular overlap, eye laterality, and a short, straight, stout beak (Martinho et al. 2014; Matsui et al. 2016; Troscianko et al. 2012). They secure tools after extracting prey by trapping it underfoot or storing it in a hole—doing so more often when tool availability is limited and the cost of tool loss is higher (Hunt 1996; Klump et al. 2015). They also use tools to explore predator models (Wimpenny et al. 2011) or to reach for food in their presence (Taylor et al. 2012).

We present a novel type of spontaneous tool use that has not previously been described in any species. We define *insert*-*and*-*transport* tool use as inserting a stick or stick-like object into another object, and carrying tool and target object away together by holding the tool only. We discuss the possible functions of this tool-use mode in the context of both the current investigation and the crows’ natural environment.

## Methods

We observed this new tool-use mode in two adult New Caledonian crows, Liane (female) and Aigaios (male). The observations were made in everyday situations and also in an unrelated study on object caching (Jacobs et al. 2014) on eight crows, in which these two subjects participated. They were wild-caught on New Caledonia as part of family groups two and a half years earlier. They were housed at the Avian Cognition Research Station associated to the Max Planck Institute for Ornithology in Seewiesen, Germany. They were housed in pairs or family groups in outdoor aviaries of between 18 and 32 m^2^ with constant access to heated and lit indoor compartments of approximately 7 m^2^. Food and water were available ad libitum.

**Fig. 1:**
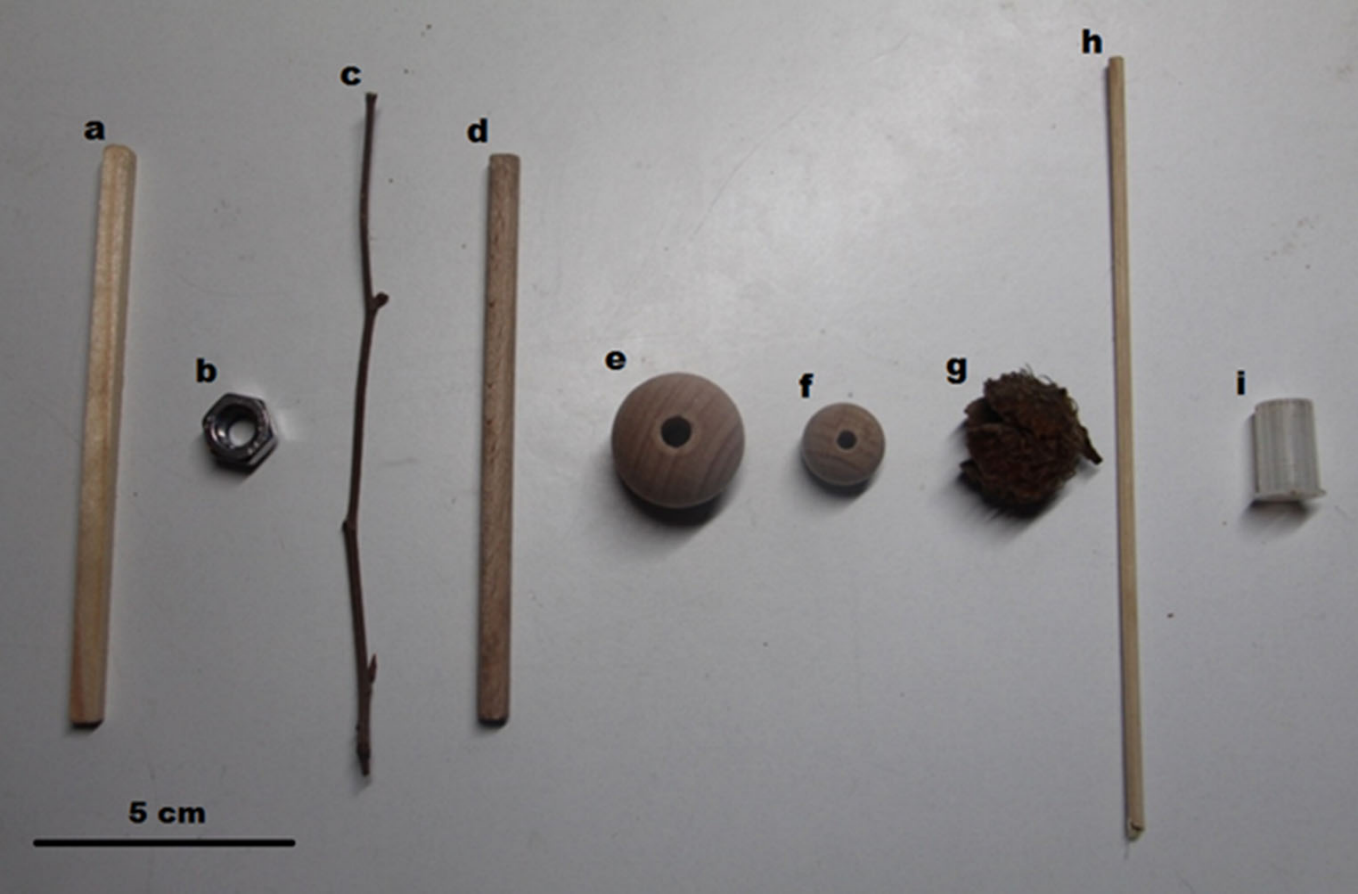
Tools and objects used for *insert*-*and*-*transport* tool use. **a** Experimental square wooden stick; **b** experimental metal nut; **c** non-experimental natural wooden stick (various types were used, but they were always thinner than the experimental sticks); **d** experimental round wooden stick; **e** experimental large wooden ball; **f** experimental small wooden ball; **g** non-experimental beech cupule (*Fagus sylvatica*); **h** non-experimental bamboo skewer; **i** non-experimental plastic cap

In the unrelated study, in which we observed some instances of the tool-use behaviour, the crows had eight 12-min trials in which they had the opportunity to interact with 16 initially novel objects, hereafter referred to as experimental tools/objects (see Fig. 1). All observations were video recorded; the recordings are available in the Electronic Supplementary Material for this paper. No statistical analyses were performed because of the low number of total occurrences.

## Results

In total, six instances of *insert*-*and*-*transport* tool use were recorded: four in the experimental and two in an everyday setting (see Table 1; ESM). Liane exhibited the behaviour in both situations. The four observations during the unrelated object-caching study involved an experimental tool twice. During none of the observations did we detect indications of stress such as approach–avoidance behaviour or sudden jumps. The subjects landed on the table voluntarily without any food incentive, and approached calmly and directly.

**Table 1:** Overview of all observations of *insert*-*and*-*transport* tool use

Observation	Date	Subject	Condition	Tool	Object
1	09-03-2013	Liane	Experimental	a	b
2	13-03-2013	Aigaios	Experimental	c	e
3	13-03-2013	Aigaios	Experimental	c	f
4	19-03-2013	Liane	Experimental	d	b
5	26-02-2014	Liane	Non-experimental	c	g
6	26-02-2014	Liane	Non-experimental	h	i

The tool and object lettering refers to Fig. 1

**Fig. 2:**
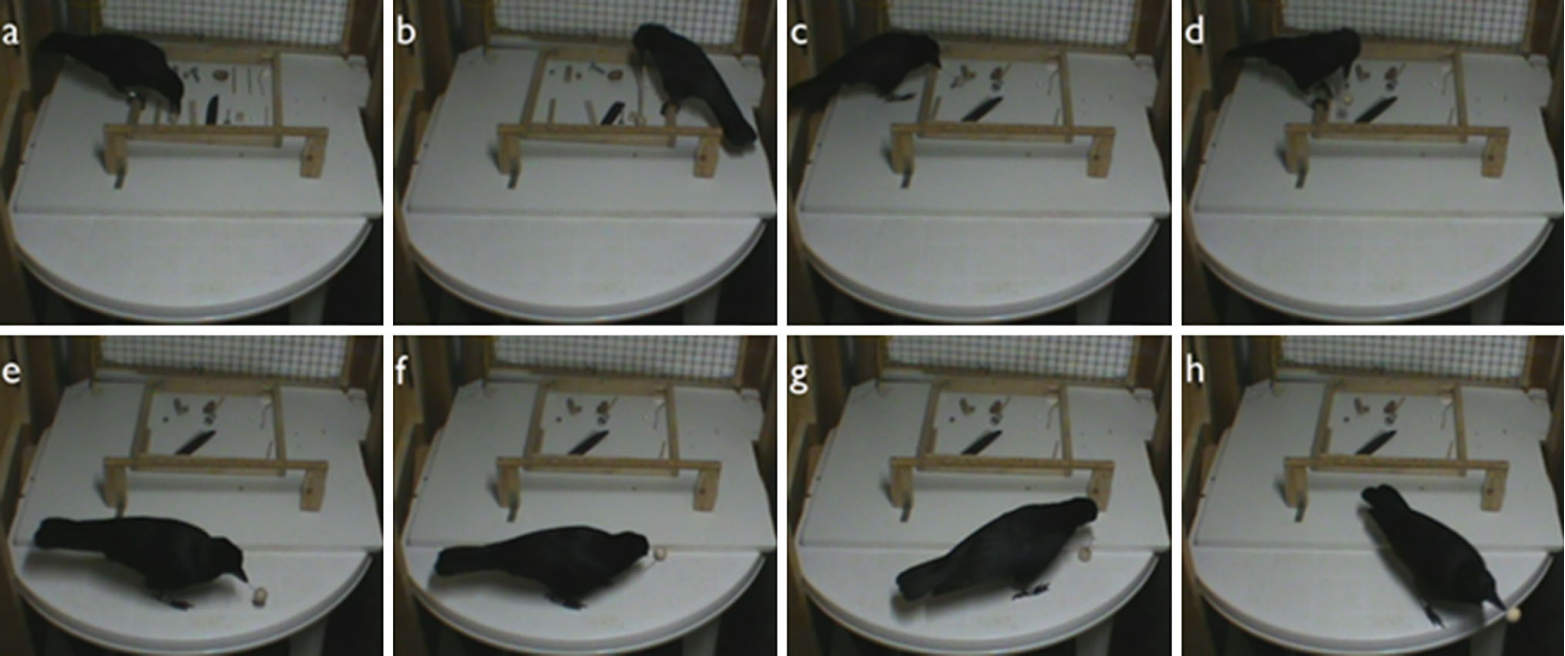
Still-frame sequence from a video showing Aigaios using a natural stick to move a large wooden ball (Observation 2; time stamps in parentheses). **a** He attempts to pick up the ball but fails (0:58); **b** he picks up an experimental stick (see Fig. 1d), fails to insert it into the hole in the ball, tries to pick up the ball with his beak but fails again, and then leaves the experimental area (1:48); **c** he returns with a thin non-experimental stick and inserts it into the ball (6:44); **d** the stick-and-ball combination allows him to lift the ball off the table (6:53); **e** he moves the combined object (6:57); **f** he puts tool and object on the table and leaves the experimental room (6:59); **g** he returns and looks down the tool into the hole in the ball (7:29); **h** he grasps the stick and leaves the experimental room, simultaneously transporting tool and target object (7:33)

Both subjects first held the target object in their beak before using the tool, with the only exception being Observation 2, where Aigaios tried but failed to pick up a large wooden ball. He initially attempted to insert an experimental tool, without success. He returned with a thinner, natural stick, inserted it into a small hole in the ball, lifted the ball using the stick, and flew off (see Fig. 2).

In Observation 6, Liane inserted a tool into a non-experimental object, which she carried and then inserted into a fold in a canvas sheet that lay on the ground. She used the tool to push the object further in. To our knowledge, this marks the first observation of New Caledonian crows using a tool for caching.

## Discussion

Two New Caledonian crows exhibited a novel tool behaviour not previously reported for any non-human species (cf. Bentley-Condit and Smith 2010; McGrew 2013; Shumaker et al. 2011): inserting a stick into an object and using the stick to transport it. The behaviour clearly differs from the *contain* mode, defined as placing “fluids or objects into or on top of another object (the tool) to control and/or transport them” (Shumaker et al. 2011, p. 14). Whereas containers can carry fluids or assemblages of very small objects, the tool reported here did not contain the object and transported only a single object. Moreover, control over the object did not appear to determine the behaviour; in most cases, the target object could be moved more effectively using the beak alone.

It is possible that the crows perceived the objects as potentially harmful and that such risk was mitigated by use of a tool. New Caledonian crows use stick tools to explore novel objects (Wimpenny et al. 2011) and avoid possible risks (Taylor et al. 2012). However, such explanations seem insufficient for our findings. In all cases, the crows had touched the objects with their beaks at least once before, so the objects were not truly novel. The crows also showed no neophobic reaction towards the objects and readily interacted with them. Neither does food search explain the behaviour. The objects in these observations had never been associated with food, and foraging techniques in the wild differ notably from our observations.

A possible functional explanation for *insert*-*and*-*transport* tool use is the simultaneous transport of both a tool and an object (or food item), which could be adaptive in the wild. New Caledonian crows often secure their tools while foraging, especially at greater heights where suitable tools become scarce (Klump et al. 2015). Although our experiment only involved non-food items, crows might target food as well: notably food with an opening that is either not immediately consumed or too large to be handled easily, such as half-opened seashells or large snails.

Subjects did not always transport the tool and object very far in our captive setting. There was also no clear reason why they would transport the object in the first place. Sometimes they brought their tool from another room, which was unnecessary if the goal was to transport the object, given that they could carry the object in their beak. In most cases, using a tool was less effective for transportation: it took time to insert the tool correctly and the tool-object combination was heavier. Given those time and energy costs, why would the crows use tools when they did not need to?

One case (Observation 2) indicated potential purposefulness of this behaviour; the subject struggled with grasping a large wooden ball and then successfully transported it with a stick. This indicates another possible function of this tool-use mode, namely control over unwieldy objects. The beak morphology of New Caledonian crows facilitates stick manipulation (Matsui et al. 2016; Troscianko et al. 2012), but it constrains handling many other objects. In the other observations, *insert*-*and*-*transport* tool use is perhaps best explained as a form of exploration or play because it was performed voluntarily in a low-stress setting without clear immediate benefit or purpose (Burghardt 2005), at least in the captive setting in which we have detected it. Playful stick handling develops into tool use in juveniles, even in the absence of demonstrators, which suggests it is an inherited action pattern (Kenward et al. 2006).

*Insert*-*and*-*transport* is a novel tool-use mode in animals as it differs notably from previously described modes. Our observations could be innovations originating in play and development without the purpose of transporting objects. Further research is needed to investigate whether it is a species-typical behaviour that might be adaptive, and controlled studies in captivity should establish the extent to which New Caledonian crows can apply this behaviour flexibly for purposes of transport.

## Electronic supplementary material

Below is the link to the electronic supplementary material.

Supplementary material 1 (M4 V 107069 kb)

## References

[CR1] Bentley-Condit VK, Smith EO (2010). Animal tool use: current definitions and an updated comprehensive catalog. Behaviour.

[CR2] Burghardt GM (2005). The genesis of animal play.

[CR3] Hunt GR (1996). Manufacture and use of hook-tools by New Caledonian crows. Nature.

[CR4] Jacobs IF, Osvath M, Osvath H, Mioduszewska B, von Bayern AM, Kacelnik A (2014). Object caching in corvids: incidence and significance. Behav Process.

[CR5] Jelbert SA, Taylor AH, Cheke LG, Clayton NS, Gray RD (2014). Using the Aesop’s fable paradigm to investigate causal understanding of water displacement by New Caledonian crows. PLoS One.

[CR6] Kenward B, Rutz C, Weir AAS, Kacelnik A (2006). Development of tool use in New Caledonian crows: inherited action patterns and social influences. Anim Behav.

[CR7] Klump BC, van der Wal JE, St Clair JJ, Rutz C (2015). Context-dependent ‘safekeeping’ of foraging tools in New Caledonian crows. Proc R Soc B.

[CR8] Martinho A, Burns ZT, von Bayern AM, Kacelnik A (2014). Monocular tool control, eye dominance, and laterality in New Caledonian crows. Curr Biol.

[CR9] Matsui H, Hunt GR, Oberhofer K, Ogihara N, McGowan KJ, Mithraratne K, Yamasaki T, Gray RD, Izawa E (2016). Adaptive bill morphology for enhanced tool manipulation in New Caledonian crows. Sci Rep.

[CR10] McGrew W (2013). Is primate tool use special? Chimpanzee and New Caledonian crow compared. Philos Trans R Soc B.

[CR11] Rutz C, Bluff LA, Reed N, Troscianko J, Newton J, Inger R, Kacelnik A, Bearhop S (2010). The ecological significance of tool use in New Caledonian crows. Science.

[CR12] Shumaker RW, Walkup KR, Beck BB (2011). Animal tool behavior: the use and manufacture of tools by animals.

[CR13] Taylor A, Hunt G, Gray R (2012). Context-dependent tool use in New Caledonian crows. Biol Lett.

[CR14] Troscianko J, Bluff LA, Rutz C (2008). Grass-stem tool use in New Caledonian crows *Corvus moneduloides*. Ardea.

[CR15] Troscianko J, von Bayern A, Chappell J, Rutz C, Martin G (2012). Extreme binocular vision and a straight bill facilitate tool use in New Caledonian crows. Nat Commun.

[CR16] von Bayern AMP, Heathcote RJP, Rutz C, Kacelnik A (2009). The role of experience in problem solving and innovative tool use in crows. Curr Biol.

[CR17] Wimpenny J, Weir A, Kacelnik A (2011). New Caledonian crows use tools for non-foraging activities. Anim Cogn.

